# Spiro-Phenylpyrazole/Fluorene as Hole-Transporting Material for Perovskite Solar Cells

**DOI:** 10.1038/s41598-017-08187-4

**Published:** 2017-08-10

**Authors:** Yang Wang, Tzu-Sen Su, Han-Yan Tsai, Tzu-Chien Wei, Yun Chi

**Affiliations:** 1 Department of Chemistry, National Tsing Hua University, Hsinchu, 30013 Taiwan; 2 Department of Chemical Engineering, National Tsing Hua University, Hsinchu, 30013 Taiwan

## Abstract

Spiro-OMeTAD with symmetric spiro-bifluorene unit has dominated the investigation of hole-transporting material (HTM) for efficient perovskite solar cells (PSCs) despite of its low intrinsic hole conductivity and instability. In this study, we designed and synthesized three asymmetric spiro-phenylpyrazole/fluorene base HTMs, namely: WY-1, WY-2 and WY-3. They exhibit excellent electrochemical properties and hole conductivities. Moreover, the PSC based on WY-1 exhibits the highest power conversion efficiency (PCE) of 14.2%, which is comparable to the control device employing spiro-OMeTAD as HTM (14.8%). These results pave the way to further optimization of both molecular design and device performance of the spiro-based HTMs.

## Introduction

Organic-inorganic lead halide perovskites have received a great deal of attention since the first report of perovskite solar cell (PSC) by Miyasaka *et al*.^1^. These materials possess many attractive merits, including strong visible absorbability^2, 3^, high carrier mobility^4, 5^ and long carrier diffusion length^6, 7^. The rational design of devices, effective modulation of interfacial contact and development of superior charge transporting materials have all contributed to the recent record-high power conversion efficiency (PCE) of 22.1%^8^ (http://www.nrel.gov/). To date, 2,2′,7,7′-tetrakis(*N*,*N*-di-*p*-methoxyphenylamine)-9,9′-spirobifluorene (spiro-OMeTAD) has been utilized as the hole-transporting materials (HTMs) in fabrication of PSCs with PCEs of over 20%^8–10^. However, spiro-OMeTAD suffers from poor hole mobility in the pristine form due to its poor crystallinity caused by the spiro structure^11, 12^. Generally speaking, there are two strategies to overcome such drawbacks^13–16^. The first method is to replace spiro skeleton with other ensembles such as pyrene^17^, 4,4-N,N′-dicarbazole-1,1′-biphenyl (CBP)^18^, quinolizino acridine^19^, functional thiophene^20, 21^, 1,1,2,2-tetraphenylethene (TPE)^22, 23^ and bifluorenylidene^24^, *etc*. for tuning the HOMO energy, hole mobility and conductivity^25–31^. The second method is to alter the spiro-bifluorene unit with the acridine/fluorene^32, 33^ or xanthene/fluorene^34, 35^ core to achieve the superior device performances.

**Figure 1:**
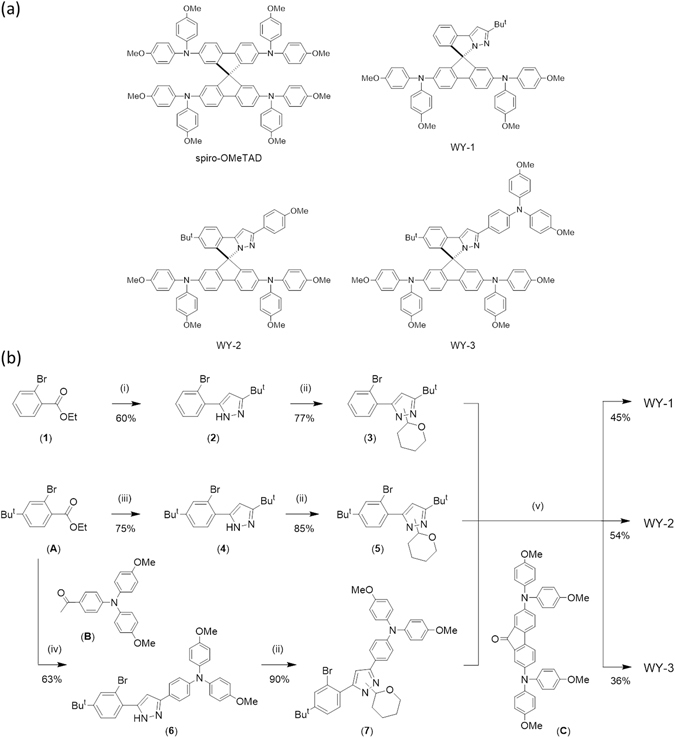
(**a**) Chemical structures of WY-1, WY-2 and WY-3 compared with Spiro-OMeTAD. (**b**) Synthetic routes for the studied HTMs.

Herein, we presented unprecedented HTMs based on spiro-phenylpyrazole/fluorene architecture; namely: WY-1, WY-2 and WY-3 (Fig. 1a). They exhibit excellent hole transporting properties as half of the molecule is constructed based on spiro-OMeTAD, while the orthogonal phenyl pyrazole fragments were fine-tuned by varying the π-conjugation and introducing substituents with distinctive steric and electronic properties^20^. Remarkably, WY-1 with the simplest structure has achieved the highest PCE of 14.2% which is comparable to spiro-OMeTAD reference (14.8%), demonstrating the advantage of simple molecular structure in achieving high efficiency PSCs.

## Results and Discussion

### Synthetic procedures

The synthetic routes of HTMs are depicted in Fig. 1b. The pyrazole intermediates were first obtained by Claisen condensation using the functional ethyl 2-bromobenzoate and acetyl compounds, followed by hydrazine cyclization in refluxing ethanol. Next, treatment of the 2-bromophenyl pyrazoles with tetrahydro-2*H*-pyran (THP) afforded a mixture of two isomers, for which the THP is located at either one of the pyrazolic nitrogen atoms. This mixture was not separated, but directly used for the subsequent reaction with n-BuLi to afford the lithiated compounds. Upon treatment with 2,7-bis(bis(4-methoxyphenyl)amino)-9*H*-fluoren-9-one in tetrahydrofuran (THF), followed by hydrolysis in a mixture of acetic acid and concentrated hydrochloric acid at room temperature, the desired spiro compounds with a variety of phenyl pyrazole fragment were obtained in high yields. All obtained compounds WY-1, WY-2 and WY-3 showed good solubility in common organic solvents, such as THF, dichloromethane (CH_2_Cl_2_), toluene and chlorobenzene, ensuring excellent processibility for fabrication of PSCs using spin coating.

### Thermal properties

Thermogravimetric analysis (TGA) and differential scanning calorimetry (DSC) measurements were recorded to study their fundamental properties (Table 1 and Figure S1). As can be seen, the decomposition temperature (T_*d*_) of WY-1, WY-2 and WY-3 were recorded to be 401 °C, 422 °C and 427 °C, respectively, which are only slightly lower than that of spiro-OMeTAD (452 °C)^35^, but are high enough to withstand the harsh condition required for the cell operation. Furthermore, the glass transition temperature (T_*g*_) increased from WY-1 (122 °C), WY-2 (136 °C) to WY-3 (145 °C) due to the higher molecular weight and skeletal rigidity. Nevertheless, all T_*g*_ are comparable to that of spiro-OMeTAD (125 °C)^35^, showing good morphological stability demanded for PSCs.

**Table 1:** Electrochemical, photophysical and thermal properties of Spiro-OMeTAD, WY-1, WY-2, and WY-3.

HTMs	λ_abs_^a^ (nm)	*E*_g,opt_^b^ (eV)	*E*_ox_^c^ (V)	HOMO^d^ (eV)	LUMO^e^ (eV)	*T*_d_ (°C)	*T*_g_ (°C)
Spiro-OMeTAD	303, 365, 386	3.00	0.002	−5.10	−2.10	452^*^	125^*^
WY-1	290, 386	2.98	0.065	−5.16	−2.18	401	122
WY-2	269, 386	2.98	0.065	−5.16	−2.18	422	136
WY-3	306, 346, 386	2.98	0.057	−5.15	−2.17	427	145

^a^Measured in THF with a concentration of 10^−5^ M. ^b^Calculated from the absorption onset. ^c^The oxidative onsets (vs FcH/FcH^+^) measured by cyclic voltammetry in CH_2_Cl_2_. ^d^HOMO = −(*E*_ox_ + 5.1) eV and LUMO = HOMO + *E*_g,opt_.

### Optical and electrochemical properties

**Figure 2:**
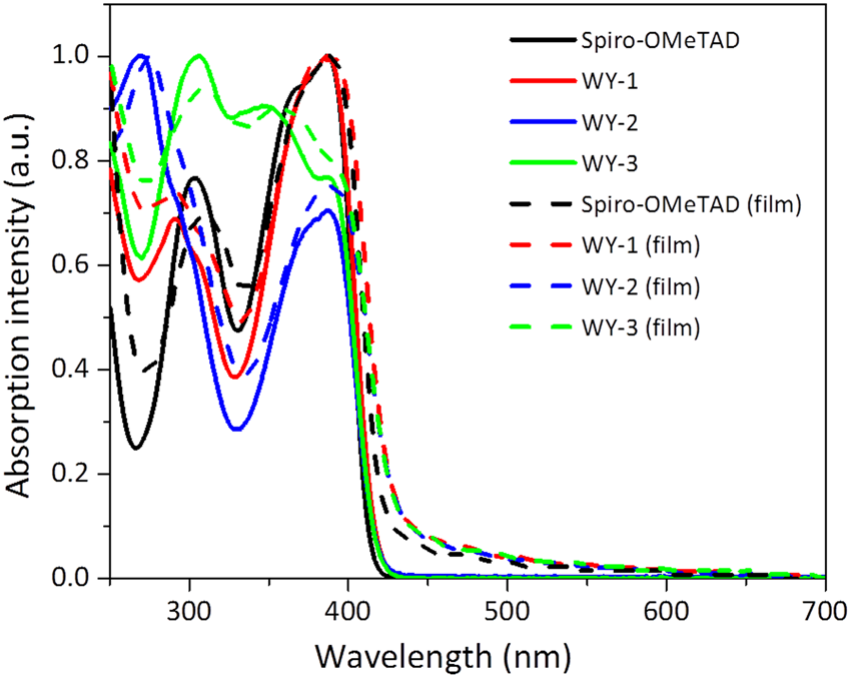
UV-vis absorption spectra of four HTMs measured in solutions (solid line) and thin films (dash line).

Figure 2 showed the UV-vis absorption of WY-1, WY-2 and WY-3 in both solution and solid states, together with those of spiro-OMeTAD for comparison, while the corresponding numerical data are depicted in Table 1. The lowest-energy absorption peak in solution occurred at 386 nm for all new HTMs and spiro-OMeTAD, which can be attributed to π−π* electronic transition at the diphenylamine substituted fluorene moiety^27^. Their optical energy gap (*E*_g,opt_), estimated from absorption onset, was calculated to be *ca*. 2.98 eV, which is identical to that of spiro-OMeTAD (3.00 eV) documented in literature^36^. Going from solution to solid state, their absorption profiles are essentially unchanged, indicating no obvious π-π stacking interaction in the solid state due to the orthogonal spiro geometry.

Cyclic voltammetry (CV) was next performed to determine the relative energy of the highest occupied molecular orbital (HOMO) (Table 1). As shown in Figure S2, all samples display reversible multi-oxidative behaviours. Particularly, the numbers of reversible CV wave are proportional to the number of diphenylamino substituents, confirming excellent electrochemical stability. In addition, their oxidative onsets versus FcH/FcH^+^ were found to be slightly more positive than spiro-OMeTAD, giving the more stabilized HOMO (−5.15 ∼ −5.16 eV) compared with spiro-OMeTAD (−5.10 eV), and were expected to have higher *V*_OC_ values for the PSCs. The stabilized HOMOs may be attributed to the reduced π-conjugation and greater electron withdrawing effect exerted by the orthogonal phenyl pyrazole. This is confirmed by theoretical calculation based on density functional theory (DFT) method using B3LYP hybrid functional and 6–31 g* basis set (Figure S3), for which the electron density of HOMO and LUMO for WY-1, WY-2 and WY-3 are all residing on both the diphenylamino substituents and adjacent fluorene unit. In sharp contrast, the frontier orbitals of spiro-OMeTAD are delocalized over both of the bifluorene cores. Such different electronic density distributions result in reduced HOMO levels for WY-1 to WY-3 which is in accordance with the experimental tendency.

### Mobility measurement

**Figure 3:**
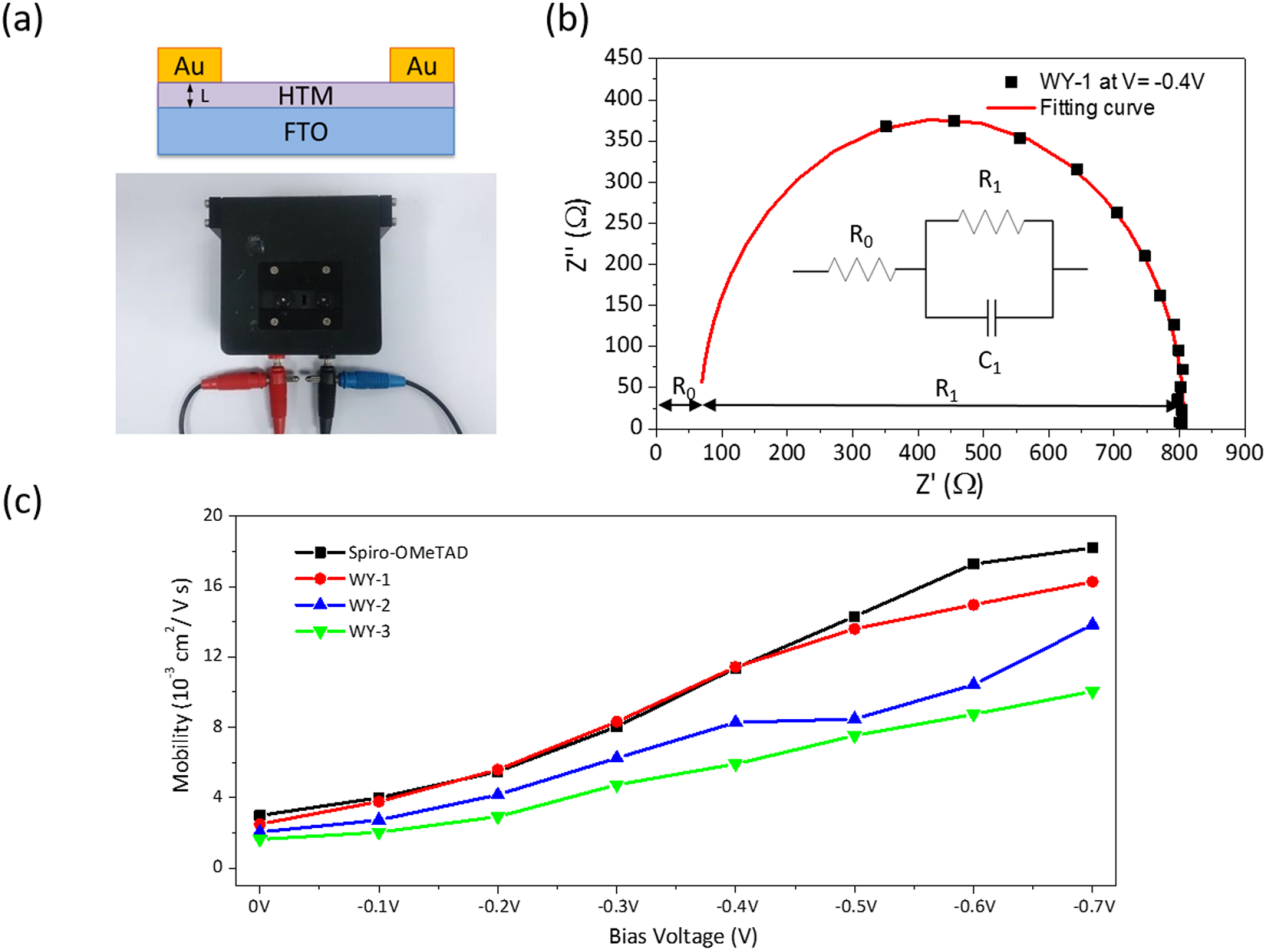
(**a**) Structural diagram of the hole-only cell Au/HTM/Au for electrochemical impedance measurement. (**b**) The typical Nyquist plot of Au/WY-1/Au measured at −0.4 V and its fitting curve using an equivalent circuit (inset). (**c**) The calculated hole mobility of HTMs vs. applied bias.

The hole mobilities were studied by measuring the electrochemical impedance measurements on a symmetrical, hole-only device comprising of Au/HTM/Au architecture (Fig. 3a) under different voltage bias over 1 MHz to 100 Hz in the dark. A typical Nyquist plot of Au/WY-1/Au measured at −0.4 V bias was shown in Fig. 3b; data points were fitted using an equivalent circuit provided in the insert of Fig. 3b. The equivalent circuit contains a series resistance (R_0_), a charge transfer resistance (R_1_) and a constant phase element (CPE_1_) which associates with charge accumulation in the depletion layer near the interface of metal electrode (gold) and semiconductor (HTM)^37, 38^. Because the HTM is an organic semiconductor itself; therefore, R_1_ and CPE_1_ decreases when forward bias increases^39^. The hole mobility could be estimated using the equation (1):

1 $$\mu =\frac{{{\rm{e}}{\rm{L}}}^{2}}{{{\rm{k}}}_{{\rm{b}}}{{\rm{T}}{\rm{R}}}_{1}{{\rm{C}}{\rm{P}}{\rm{E}}}_{1}}$$

where L is the width of the depletion^40, 41^ zone and is approximately equal to the film thickness at such resistive and thin domain, k_b_ is Boltzmann constant, and T is the absolute temperature. CPE_1_ and R_1_ were obtained by curve fitting. The relationship of hole mobility with different applied bias is shown in Fig. 3c. It can be seen that WY-1 has identical mobility to spiro-OMeTAD except at high bias over 0.5 V, while the mobilities of WY-2 and WY-3 are both lower than WY-1 and spiro-OMeTAD. The lower mobility for WY-2 and WY-3 may originate from their greater size and higher rigidity^33^, both are expected to jeopardize the hole transporting property.

### Performance of Perovskite solar cells

**Figure 4:**
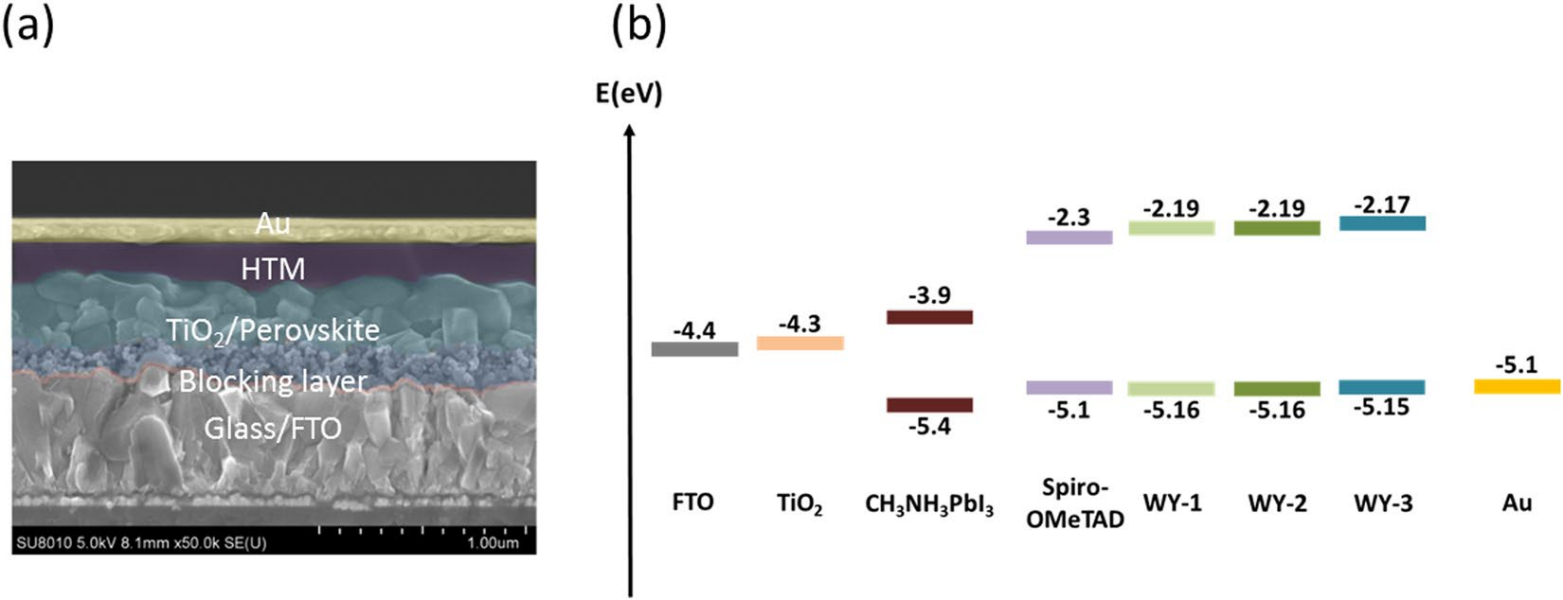
(**a**) Cross-section view of representative PSC and (**b**) energy level diagram of the materials used in the studied PSCs (right).

The procedures of perovskite solar cell (PSC) fabrication are detailed in the experimental section. The PSC features a multi-layered structure of FTO/TiO_2_ compact layer/ mesoporous TiO_2_ scaffold/ perovskite/ HTM/ Au (Fig. 4a). The perovskite layer (CH_3_NH_3_PbI_3_) was sequentially deposited, following the method developed by Burschka *et al*.^42^ Fig. 4b reveals that the energy levels of new HTMs are suitable for making efficient PSCs. HTMs were spin-coated with typical additives such as bis(trifluoromethane) sulfonimide lithium salt (Li-TFSI) and 4-tert-butylpyridine (tBP) (detailed information can be found in the experimental section).

**Figure 5:**
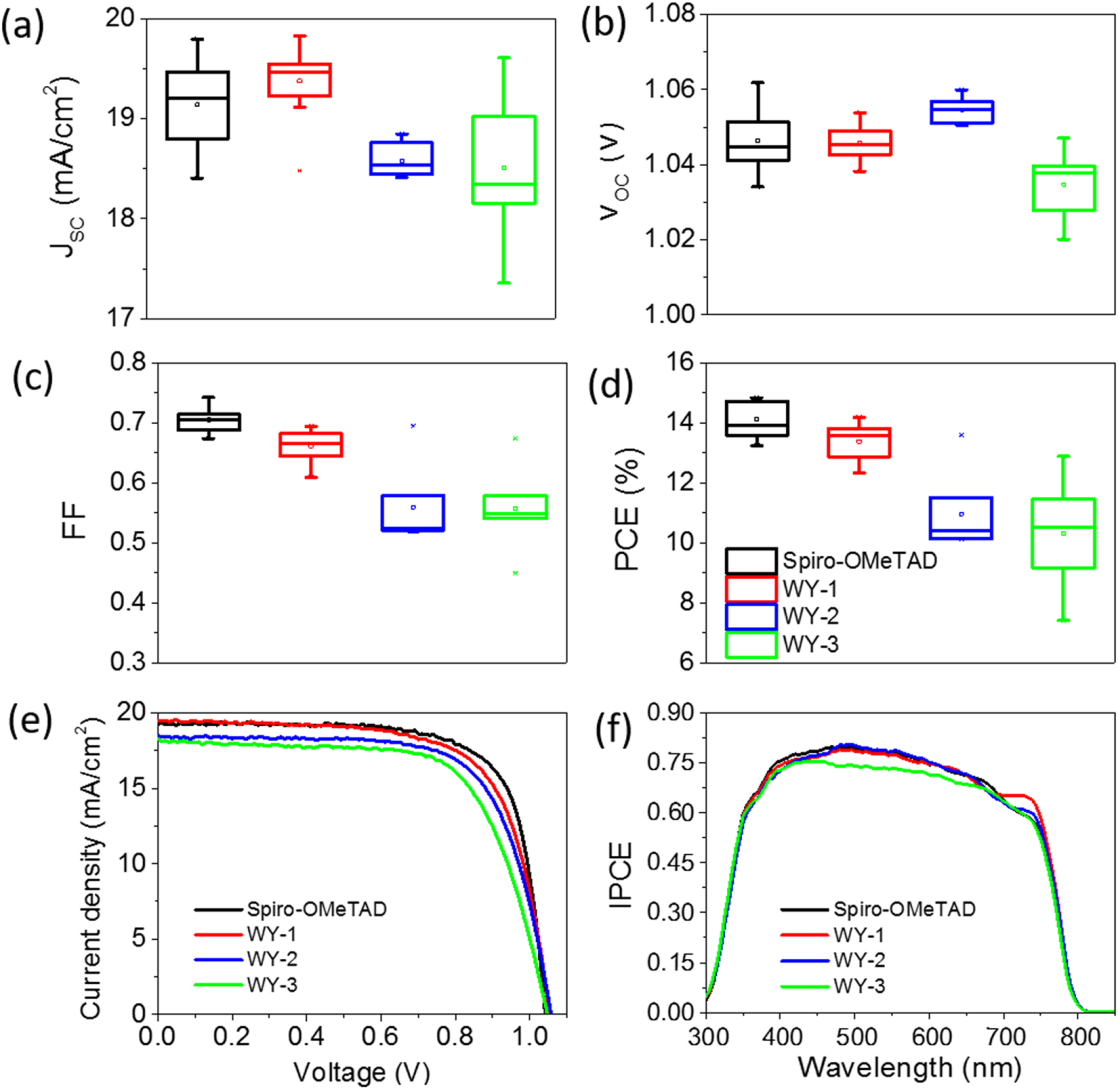
(**a**–**d**) Averaged J_SC_, V_OC_, FF and PCE of PSCs employed with different HTMs. (**e**) I-V curves of best-performing PSCs employing the studied HTMs, measured under AM 1.5 G illumination, 100 mW cm^−2^. (**f**) IPCE diagrams of the corresponding devices; the total currents derived from the integrated curves are: 18.05 mA/cm^2^, 18.16 mA/cm^2^, 18.08 mA/cm^2^ and 17.32 mA/cm^2^ for the spiro-OMeTAD, WY-1, WY-2, and WY-3-based device, respectively.

The photovoltaic performances of PSCs made with all studied HTMs and benchmark spiro-OMeTAD are presented in Fig. 5. Figure 5a–d summarizes photovoltaic performance of multiple devices from different batches using the same recipe and under 100 mW cm^−2^ AM1.5 G solar illumination. It can be seen that WY-1 exhibits competitive PCE vs. spiro-OMeTAD, while WY-2 and WY-3 show lowered short-circuit current density (J_SC_) and fill factor (FF), resulting in slightly inferior PCE. Figure 5e depicts the I-V curves of the best devices based on new HTMs and spiro-OMeTAD, while corresponding I-V parameters are listed in Table 2. The PSC using WY-1 achieves a J_SC_ of 19.48 mA/cm^2^, an open-circuit voltage (V_OC_) of 1.05 V, a fill factor (FF) of 0.69, and a PCE of 14.20%, respectively. Under the same fabrication conditions, the benchmark spiro-OMeTAD-based device exhibits J_SC_ of 19.20 mA/cm^2^, V_OC_ of 1.04 V, FF of 0.74 and PCE of 14.84%. From the incident photon to current efficiency (IPCE) provided in Fig. 5f, the higher J_SC_ obtained in WY-1-based device is derived from its higher IPCE at 700 to 750 nm, which may be due to the superior optical transmittance of WY-1 at around 700 nm (Figure S4), allowing better IPCE over that of spiro-OMeTAD. But owing to the lowered hole mobility vs. spiro-OMeTAD, WY-1 device suffers from notable ohmic losses in FF due to the slightly higher resistivity. In addition, WY-2 and WY-3 based PSCs exhibit very similar photovoltaic performance with J_SC_ of 18.50 and 18.27 mA cm^−2^, V_OC_ of 1.06 and 1.05 V, FF of 0.70 and 0.67 and PCE of 13.60 and 12.89%, respectively. The inferior performance of WY-2 and WY-3 than WY-1 may be attributed to the larger molecular volume vs. WY-1. Hence, the greater steric hindrance in WY-2 and WY-3 renders poor intermolecular π-stacking interaction (i.e. carrier transport) in the deposited thin film and thus lowers the J_SC_ and FF of the PSC.

**Table 2:** Photovoltaic parameters of PSCs with different hole transporting layer. Photomasks (0.2 × 0.5 cm^2^) made of thin metal sheet were applied before all measurements.

Type of BL		J_SC_ (mA/cm2)	V_OC_ (mV)	FF	PCE (%)	R_s_ (Ω)	HI
Best performing	Spiro-OMeTAD	19.20	1.041	0.742	14.84	32.47	0.04
	WY-1	19.48	1.053	0.691	14.19	53.52	0.07
	WY-2	18.50	1.057	0.696	13.60	63.10	0.08
	WY-3	18.27	1.047	0.674	12.89	85.45	0.05
Average*	Spiro-OMeTAD	19.14 ± 0.46	1.046 ± 0.007	0.705 ± 0.022	14.13 ± 0.57	47.96 ± 11.86	0.09 ± 0.07
	WY-1	19.38 ± 0.33	1.046 ± 0.005	0.662 ± 0.025	13.36 ± 0.58	51.64 ± 13.09	0.09 ± 0.04
	WY-2	18.58 ± 0.15	1.055 ± 0.003	0.560 ± 0.059	10.96 ± 1.17	89.89 ± 12.57	0.39 ± 0.13
	WY-3	18.50 ± 0.65	1.035 ± 0.003	0.556 ± 0.061	10.32 ± 1.72	100.10 ± 34.37	0.35 ± 0.16

The hysteresis index (HI) is defined as (P_max(forward scan)_/P_max(backward scan)_)−1, which P_max_ is the maximum power in I-V measurement.

*These values were obtained from 6–8 devices.

## Conclusions

In summary, we reported three HTMs with asymmetric spiro-phenylpyrazole/fluorene architecture, which renders the effective modification on conventional symmetric spiro-OMeTAD. These new HTMs possess suitable HOMO level which could be beneficial to the high V_OC_. Moreover, they exhibit adequate thermal, optical and electrochemical properties but a slightly different hole-transporting ability, while WY-1 shows the best hole mobility relative to that of WY-2 and WY-3. As a result, WY-1 based device exhibits the highest PCE of 14.20% with a J_SC_ of 19.48 mA/cm^2^, a V_OC_ of 1.05 V and a FF of 0.69 which are very close to the benchmark HTM spiro-OMeTAD (14.84%). On the other hand, higher series resistances originate from lowered hole mobility are found in these WY-based PSCs, rendering lowered J_SC_ and FF. Currently, modification on molecular structure and optimization on concentration of additives are undergoing in our laboratory to solve this issue. We believe that further modulation on such spiro-phenylpyrazole/fluorene structure will push the device performance to an even higher level.

## Methods

### General Information and Materials

All reactions were performed under a nitrogen atmosphere and solvents were distilled from appropriate drying agents prior to use. Commercially available reagents were used without purification unless otherwise stated. The starting materials (A)^43^, (B)^44^ and (C)^33^ were prepared according to the literature methods.

### Synthetic procedures

Experimental conditions: (i) 3,3-dimethylbutan-2-one, NaH, THF, reflux; then N_2_H_4_·H_2_O, EtOH, reflux; (ii) 3,4-dihydro-2*H*-pyran, CH_2_Cl_2_, reflux; (iii) 1-(4-methoxyphenyl)ethanone, NaH, THF, reflux; then N_2_H_4_·H_2_O, EtOH, reflux; (iv) NaH, THF, reflux; then N_2_H_4_·H_2_O, EtOH, reflux; (v) *n*-BuLi, THF, −78 °C to RT; then HCl, AcOH, reflux.

#### 3-*Tert*-butyl-5-(2-bromophenyl)-1*H*-pyrazole (2)

NaH (478 mg, 19 mmol) was suspended in 16 mL of dry THF under nitrogen, and was added dropwise 3,3-dimethylbutan-2-one (1.27 g, 12.6 mmol) dissolved in 8 mL dry THF at 0 °C. The mixture was stirred for 1 h at RT, and ethyl 2-bromobenzoate (**1**, 4.38 g, 18.7 mmol) dissolved in 16 mL dry THF was added dropwise at 0 °C. The mixture was first refluxed for 4 h, and it was poured into a mixture of water and ethyl acetate and neutralized with 2 M HCl_(aq)_. The organic layer was washed with brine and water, and dried over anhydrous Na_2_SO_4_. The crude product was obtained by concentrating the solution to dryness. Then, this product was dissolved in EtOH (65 mL), and reacted with hydrazine hydrate (3 mL, 63 mmol) in presence of *p*-toluenesulfonic acid monohydrate (228 mg, 1.2 mmol) at reflux for 12 h. After then, the solution was evaporated to dryness and the residue dissolved in ethyl acetate. The organic layer was washed with NaHCO_3_ solution, brine and water in sequence, and dried over anhydrous Na_2_SO_4_. The pure product can be obtained by column chromatography eluting with a mixture of hexane: EA = 4:1. Overall yield: 60%. ^1^H NMR (400 MHz, CDCl_3_): δ 7.63 (d, *J* = 8.0 Hz, 1 H), 7.59 (d, *J* = 7.8 Hz, 1 H), 7.31 (t, *J* = 7.5 Hz, 1 H), 7.16 (t, *J* = 8.4 Hz, 1 H), 6.48 (s, 1 H), 1.33 (s, 9 H).

#### 3-*Tert*-butyl-5-(2-bromophenyl)-1-(tetrahydro-2*H*-pyran-2-yl)-1*H*-pyrazole (3)

A mixture of compound **2** (2.09 g, 7.5 mmol), 3,4-dihydro-2*H*-pyran (2.52 g, 30 mmol), *p*-toluenesulfonic acid monohydrate (140 mg, 0.75 mmol) and CH_2_Cl_2_ (50 mL) was refluxed overnight under nitrogen. The mixture was washed with NaHCO_3_, brine and water and dried over anhydrous Na_2_SO_4_. The solid was next purified by column chromatography using hexane: EA = 20:1 as the eluent. Yield: 77%. ^1^H NMR (400 MHz, CDCl_3_): δ 7.65 (dd, *J* = 7.8, 1.1 Hz, 1 H), 7.43 (dd, *J* = 7.6, 1.7 Hz, 1 H), 7.35 (td, *J* = 7.5, 1.3 Hz, 1 H), 7.27–7.22 (m, 1 H), 6.16 (s, 1 H), 4.93–4.87 (m, 1 H), 4.00–3.96 (m, 1 H), 3.39–3.33 (m, 1 H), 2.46–2.40 (m, 1 H), 2.03–1.99 (m, 1 H), 1.88–1.83 (m, 2 H), 1.67–1.64 (m, 1 H), 1.45–1.41 (m, 1 H), 1.33 (s, 9 H).

#### 5-(4-*Tert*-butyl-2-bromophenyl)-3-(4-methoxyphenyl)-1*H*-pyrazole (4)

Following the same procedure described for **2**, treatment of **A** and dimethylbutan-2-one afforded a white product **4** in 75% yield. ^1^H NMR (400 MHz, CDCl_3_): δ 7.69 (d, *J* = 8.8 Hz, 2 H), 7.66 (d, *J* = 1.9 Hz, 1 H), 7.52 (d, *J* = 8.1 Hz, 1 H), 7.38 (dd, *J* = 8.2, 1.9 Hz, 1 H), 6.95 (d, *J* = 8.8 Hz, 2 H), 6.82 (s, 1 H), 3.84 (s, 3 H), 1.33 (s, 9 H).

#### 5-(4-*Tert*-butyl-2-bromophenyl)-1-(tetrahydro-2*H*-pyran-2-yl)-3-(4-methoxyphen-yl)-1*H*-pyrazole (5)

Following the procedure described for **3**, combination of **4** with 3,4-dihydro-2*H*-pyran afforded a white product **5** in 85% yield. ^1^H NMR (400 MHz, CDCl_3_): δ 7.80 (d, *J* = 8.9 Hz, 2 H), 7.68–7.67 (m, 1 H), 7.39 (d, *J* = 1.1 Hz, 2 H), 6.91 (d, *J* = 8.9 Hz, 2 H), 6.53 (s, 1 H), 4.98–4.96 (m, 1 H), 4.04–4.01 (m, 1 H), 3.82 (s, 3 H), 3.46–3.40 (m, 1 H), 2.59–2.57 (m, 1 H), 2.10–2.05 (m, 1 H), 1.97–1.93 (m, 1 H), 1.74–1.68 (m, 2 H), 1.63–1.60 (m, 1 H), 1.35 (s, 9 H). MS [FAB], m/z 470.2, M^+^.

#### *N*-(4-(5-(4-*Tert*-butyl-2-bromophenyl)-1*H*-pyrazol-3-yl)phenyl)-4-methoxy-*N*-(4-methoxyphenyl)benzenamine (6)

Following the procedure described for **2**, condensation of compounds **A** and **B** afforded a white product **6** in 63% yield. ^1^H NMR (400 MHz, acetone-*d*_6_): δ 7.71 (d, *J* = 1.9 Hz, 1 H), 7.68–7.64 (m, 3 H), 7.49 (dd, *J* = 8.2, 2.0 Hz, 1 H), 7.07–6.90 (m, 11 H), 3.78 (s, 6 H), 1.34 (s, 9 H).

#### *N*-(4-(5-(4-*Tert*-butyl-2-bromophenyl)-1-(tetrahydro-2*H*-pyran-2-yl)-1*H*-pyrazol-3-yl)phenyl)-4-methoxy-*N*-(4-methoxyphenyl)benzenamine (7)

Following the procedure described for **3**, treatment of **6** with 3,4-dihydro-2*H*-pyran afforded a white product **7** in 90% yield. ^1^H NMR (400 MHz, CDCl_3_): δ 7.66 (d, *J* = 7.5 Hz, 1 H), 7.50–7.46 (m, 3 H), 7.39–7.33 (m, 1 H), 7.11 (d, *J* = 8.7 Hz, 1 H), 7.05–7.03 (m, 3 H), 6.94 (d, *J* = 7.5 Hz, 2 H), 6.86–6.79 (m, 4 H), 6.50 (s, 1 H), 5.23–5.20 (m, 1 H), 4.17–4.13 (m, 1 H), 3.78 (s, 6 H), 3.64–3.60 (m, 1 H), 2.69–2.62 (m, 1 H), 2.08–2.04 (m, 1 H), 1.84–1.72 (m, 4 H). MS [FAB], m/z 667.3, M^+^.

#### Compound WY-1

A solution of compound **3** (433 mg, 1.22 mmol) in dry THF (5 mL) was treated with *n*-BuLi (731 μL, 2.5 M in n-hexane) under nitrogen at −78 °C. After 30 min, a solution of compound **C** (551 mg, 0.87 mmol) in THF (5 mL) was added dropwise. The mixture was stirred for 30 min at −78 °C, and allowed to warm up to RT. After 12 h, the solution was concentrated and the residue was extracted with CH_2_Cl_2_ and washed with brine and water in sequence and finally dried over anhydrous Na_2_SO_4_. The alcohol intermediate was obtained by column chromatography using hexane: EA = 4:1 as the eluent. It was next added to a mixture of concentrated HCl solution (1.5 mL) and acetic acid (20 mL). After stirring for 1 h at RT, the mixture was quenched with ice water and neutralized with NaHCO_3_(aq). The crude product was extracted with CH_2_Cl_2_ and washed with water. The organic layer was dried over anhydrous Na_2_SO_4_ and purified by silica gel column chromatography eluting with a mixture of hexane: EA = 8:1, giving a pale green product in 45% yield. ^1^H NMR (400 MHz, DMSO-*d*_6_): δ 7.59–7.56 (m, 3 H), 7.34 (t, *J* = 7.6 Hz, 1 H), 7.17 (t, *J* = 7.6 Hz, 1 H), 6.81–6.72 (m, 19 H), 6.33 (s, 1 H), 6.00 (s, 2 H), 3.64 (s, 12 H), 1.19 (s, 9 H). ^13^C NMR (101 MHz, acetone): δ 167.06, 155.87, 148.04, 147.87, 146.08, 145.74, 140.52, 134.01, 130.64, 128.46, 127.55, 126.15, 122.62, 121.45, 120.22, 120.20, 115.65, 114.56, 92.99, 76.33, 54.70, 32.60, 30.21. MS [FAB], m/z 816.4, M^+^. Anal. Calcd. for C_54_H_48_N_4_O_4_: C, 79.39; H, 5.92; N, 6.86. Found: C, 78.52; H, 5.90; N, 6.16.

#### Compound WY-2

Following the procedure described for **WY-1**, treatment of **C** with **5** afforded a pale yellow product in 54% yield. ^1^H NMR (400 MHz, DMSO-*d*_6_): δ 7.65–7.56 (m, 5 H), 7.44 (d, *J* = 8.0 Hz, 1 H), 6.92 (d, *J* = 12.9 Hz, 2 H), 6.79–6.74 (m, 12 H), 6.66–6.64 (m, 9 H), 6.10 (s, 1 H), 3.74 (s, 3 H), 3.60 (s, 12 H), 1.18 (s, 9 H). ^13^C NMR (101 MHz, acetone): δ 159.44, 156.11, 155.90, 151.48, 148.07, 147.57, 147.10, 145.96, 140.53, 134.26, 127.92, 127.10, 126.53, 125.96, 125.56, 124.82, 121.74, 120.31, 120.04, 119.63, 116.36, 114.49, 113.80, 93.04, 76.63, 54.68, 34.74, 30.57. MS [FAB], m/z 922.5, M^+^. Anal. Calcd. for C_61_H_54_N_4_O_5_: C, 79.37; H, 5.90; N, 6.07. Found: C, 78.66; H, 5.91; N, 6.14.

#### Compound WY-3

Following the procedure described for **WY-1**, treatment of **C** with **7** afforded a pale yellow product in 36% yield. ^1^H NMR (400 MHz, DMSO-*d*_6_): δ 7.58 (d, *J* = 8.3 Hz, 2 H), 7.56–7.51 (m, 3 H), 7.42 (dd, *J* = 8.1, 1.6 Hz, 1 H), 6.97–6.96 (m, 4 H), 6.88–6.85 (m, 4 H), 6.78–6.72 (m, 15 H), 6.66–6.63 (m, 8 H), 6.09 (s, 1 H), 3.70 (s, 6 H), 3.60 (s, 12 H), 1.16 (s, 9 H). ^13^C NMR (101 MHz, acetone): δ 156.22, 156.03, 155.83, 151.35, 148.21, 148.10, 147.60, 147.08, 146.13, 140.74, 140.57, 134.32, 127.93, 126.68, 126.45, 126.04, 125.93, 125.67, 121.98, 120.34, 120.21, 120.18, 119.49, 116.46, 114.68, 114.51, 93.06, 76.82, 54.82, 54.71, 34.66, 30.78. MS [FAB], m/z 1119.5, M^+^. Anal. Calcd. for C_74_H_65_N_5_O_6_: C, 79.33; H, 6.25; N, 5.85. Found: C, 78.50; H, 6.24; N, 5.89.

### Fabrication of perovskite solar cells

FTO glass substrates (2.2 mm, 8 Ω/□, NSG, Japan) were etched by a laser engraver (LMF-020F, Taiwan) to obtain the required electrode patterns. The etched sheets were then cleaned by commercial detergent solution (PK-LCG46, USA) and DI-water in sequence under ultrasonic bath for 30 minutes. The electrodeposited TiO_2_ blocking layer (ED-BL) was anodically electrodeposited on clean FTO in an aqueous solution containing 250 mM TiCl_4_ (20% in 2 N HCl, Acros Organics) for 300 seconds^45^. Subsequently, 200 nm-thick mesoporous TiO_2_ layer was deposited by spin-coating commercial TiO_2_ paste (30 nm, G24 power Ltd. United Kingdom) diluted in anhydrous ethanol by a 1:5 weight ratio at 6000 rpm for 30 seconds, followed by heating at 120 °C and 500 °C for 5 minutes and 30 minutes, respectively. The resultant film was treated in a 40 mM TiCl_4_ aqueous solution at 70 °C for 30 minutes and followed by annealing at 450 °C for 30 minutes. After cooling down to RT, the perovskite layer was applied following the published sequential deposition method^42^. In brief, 1.2 M PbI_2_ (99.999%, Sigma-Aldrich) was dissolved in a mixture of DMF (N,N-dimethylformamide, 99.8%, Merck) and DMSO (Dimethyl sulfoxide, 99.9%, J. T. Baker). Next, the ED-BL coated TiO_2_ scaffold was infiltrated by PbI_2_ solution by spin-coating at 6000 rpm for 10 seconds and followed by heating at 70 °C for 30 minutes. The substrates were immersed into a solution containing 63 mM methylamine iodide (MAI)^46^ in 2-propanol for 1 minute, then dried and baked on 70 °C for 20 minutes. Finally, the hole-transporting layer was deposited on top of the perovskite layer by spin-coating at 3000 rpm for 30 seconds using of HTM solution, which contained 75 mM of the studied HTM, 20 mM of bis(trifluoromethane)sulfonimide lithium salt (Li-TFSI, Sigma-Aldrich) and 120 mM of 4-tert-butylpyridine (TBP, 96%, Sigma-Aldrich) in chlorobenzene. After storing the device at dry atmosphere overnight, a gold metal film was thermally evaporated onto HTM layer as the back electrode.

### Characterization and measurement

The ^1^H NMR spectra were measured with a Varian Mercury-400 instrument. Elemental analysis was carried out on a Heraeus CHN-O Rapid Elementary Analyzer. Mass spectra were recorded on a JEOL SX-102A instrument operating in electron impact (EI) or fast atom bombardment (FAB) mode. The thermogravimetric analysis and differential scanning calorimetry were measured on a Seiko SSC 5000 instrument. UV-Vis spectra were recorded on a Hitachi U-3900 spectrophotometer. Cyclic voltammetry was conducted on a CHI 621 A Electrochemical Analyzer with a conventional three-electrode system consisting of a platinum working electrode, a platinum counter electrode and an Ag/AgCl reference electrode, and using 0.1 M tetrabutylammonium hexafluorophosphate (TBAPF_6_) as the supporting electrolyte. All potentials were calibrated against the ferrocene/ferrocenium couple assuming FcH/FcH^+^  = −5.1 eV^47^. Cross-sectional view of PSC was investigated using a Hitachi SU-8010 field emission scanning electron microscope. The conductivity of HTM was investigated by a computer-controlled Autolab PGSTAT30 Potentiostat/Galvanostat. The I-V characteristic was measured using a Keithley 2400 digital source meter under AM1.5 G illumination at intensity of 100 mW/cm^2^ (Peccell Technologies, PEC-L15). A KG3 monocrystalline silicon photodiode (Oriel, USA) was used to calibrate the light intensity. A 0.2 × 0.5 cm^2^ photo-mask was attached to the front side of the PSC to accurately control the illuminating area. The IPCE was measured using monochromatic light illumination (Peccell Technologies, PEC-20).

## Electronic supplementary material

Supplementary Information of Spiro-Phenylpyrazole/Fluorene as Hole-Transporting Material for Perovskite Solar Cells
