# Supplementary material for: Spiro-Phenylpyrazole/Fluorene as Hole-Transporting Material for Perovskite Solar Cells
Source: Sci Rep. 2017 Aug 10;7:7859. doi: 10.1038/s41598-017-08187-4 (PMC5552831; doi:10.1038/s41598-017-08187-4)
Supplement: Supplementary file 1 — Supplementary Information of Spiro-Phenylpyrazole/Fluorene as Hole-Transporting Material for Perovskite Solar Cells [file 41598_2017_8187_MOESM1_ESM.pdf]

Supplementary Information of

Spiro-Phenylpyrazole/Fluorene as Hole-Transporting Material for Perovskite Solar Cells

Yang Wang‡[1], Tzu-Sen Su ‡[2], Han-Yan Tsai[1], Tzu-Chien Wei [2]* and Yun Chi[1]*

[1]Department of Chemistry, National Tsing Hua University, Hsinchu 30013, Taiwan.

[2]Department of Chemical Engineering, National Tsing Hua University, Hsinchu 30013, Taiwan.

‡ The first two authors have contributed equally to this work

*Corresponding author: Tzu-Chien Wei

Tel: +886-3-5715131 ext.33669

E-mail: tcwei@mx.nthu.edu.tw

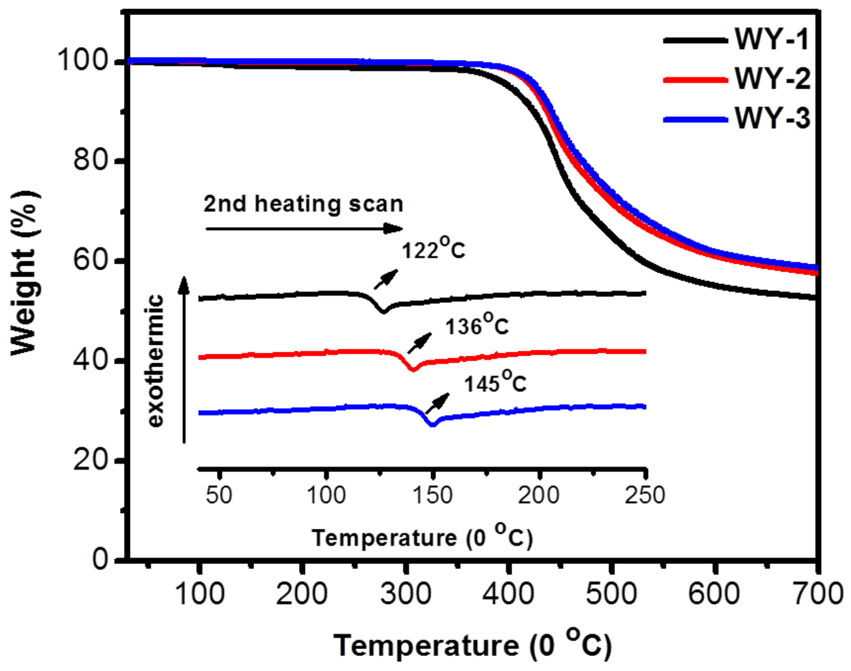

Figure S1. Thermogravimetric analysis (TGA) spectra and differential scanning calorimetry (DSC) second heating curves (inserted) of HTMs.

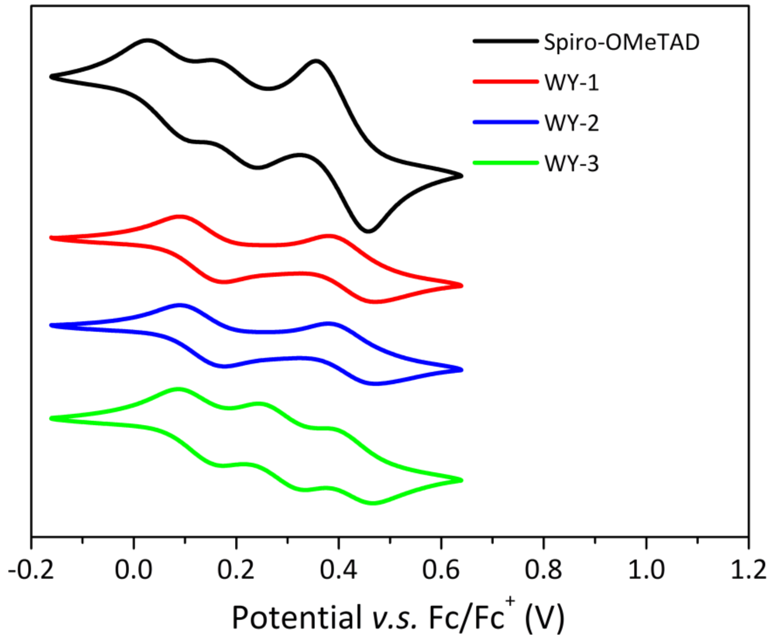

Figure S2. CV curves of four HTMs versus FcH/FcH^+ measured in CH_2Cl_2 solution.

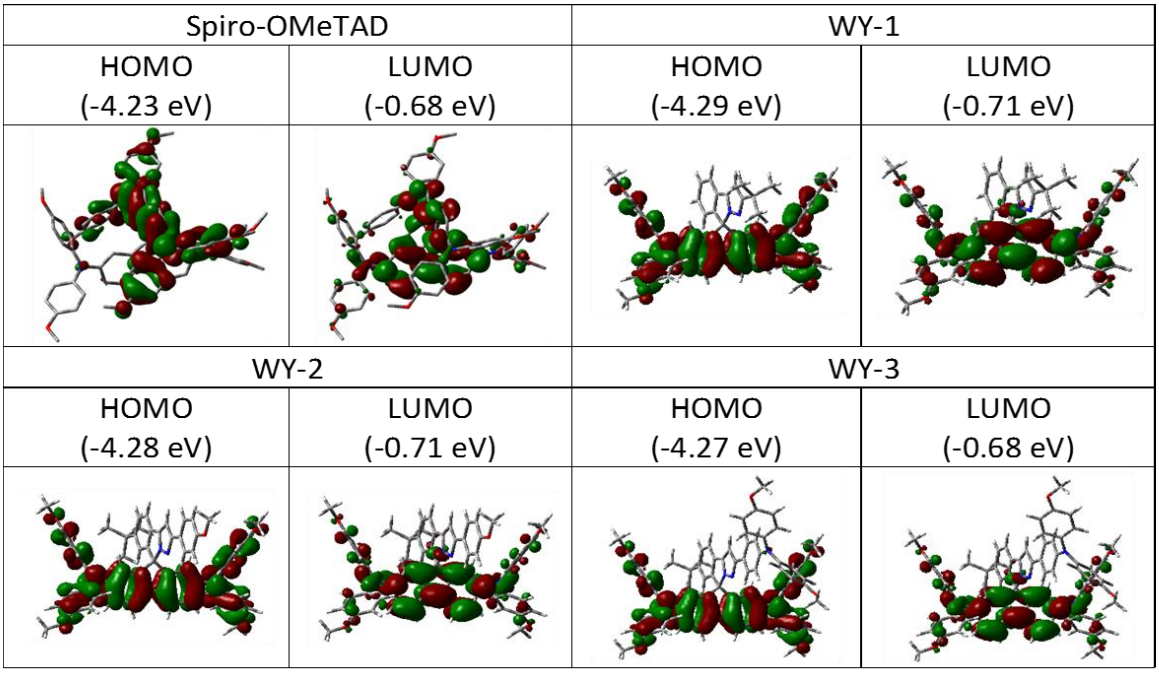

Figure S3. The calculated spatial distributions of HOMOs and LUMOs of HTMs.

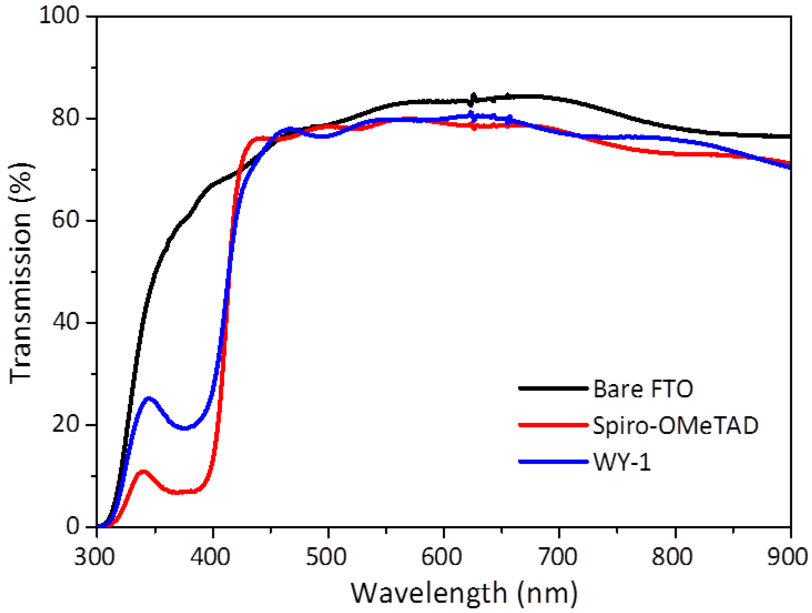

Figure S4. The transmission spectra of the bare FTO, Spiro-OMeTAD and WY-1.

(a)

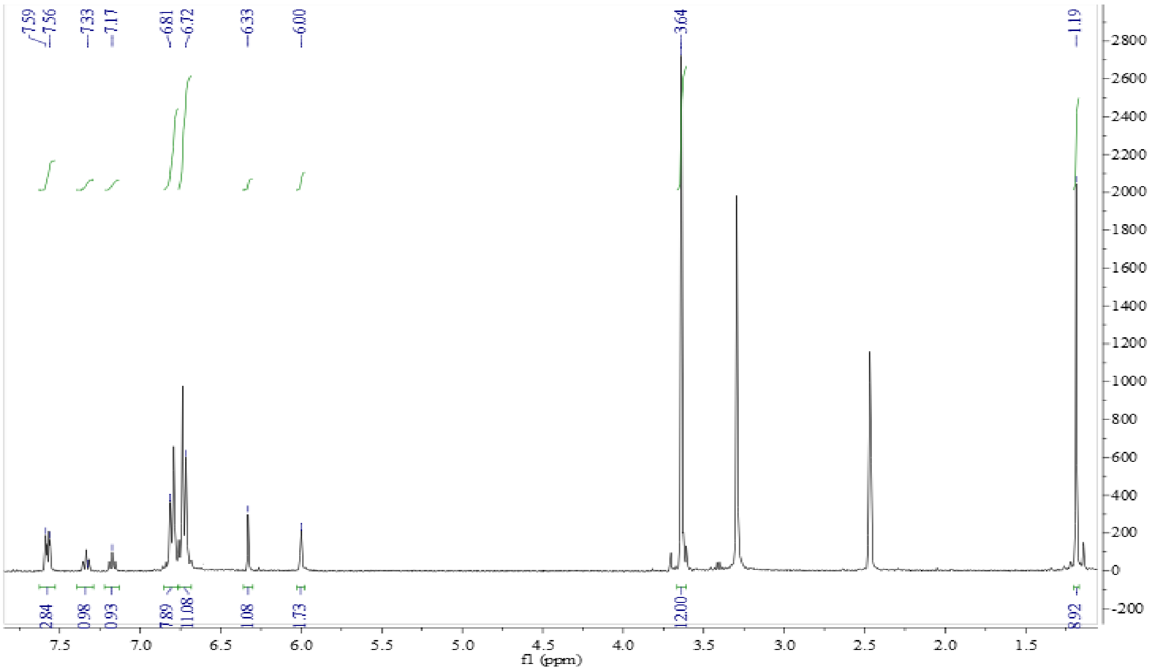

(b)

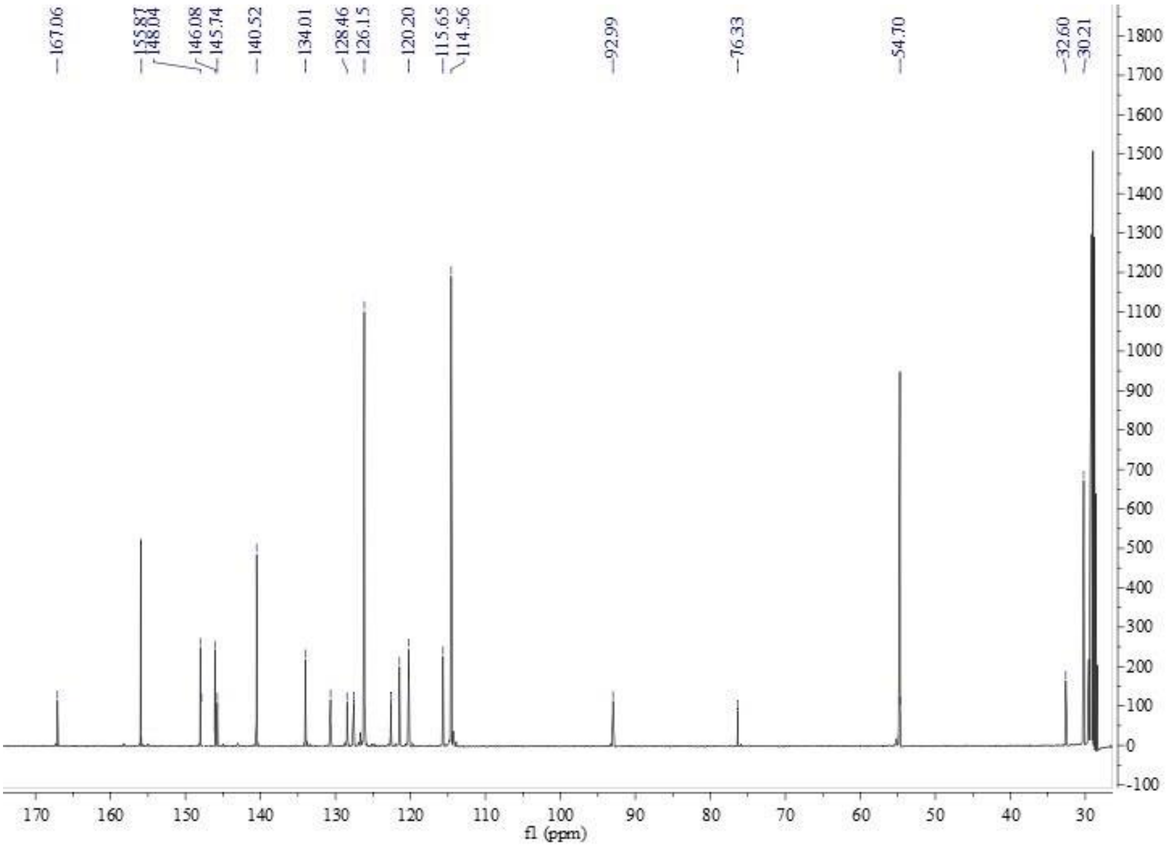

Figure S5. (a)^{1}H-NMR spectrum and (b)^{13}C-NMR spectrum of WY-1 recorded in DMSO-d_6 and acetone-d_6 at RT, respectively.

(a)

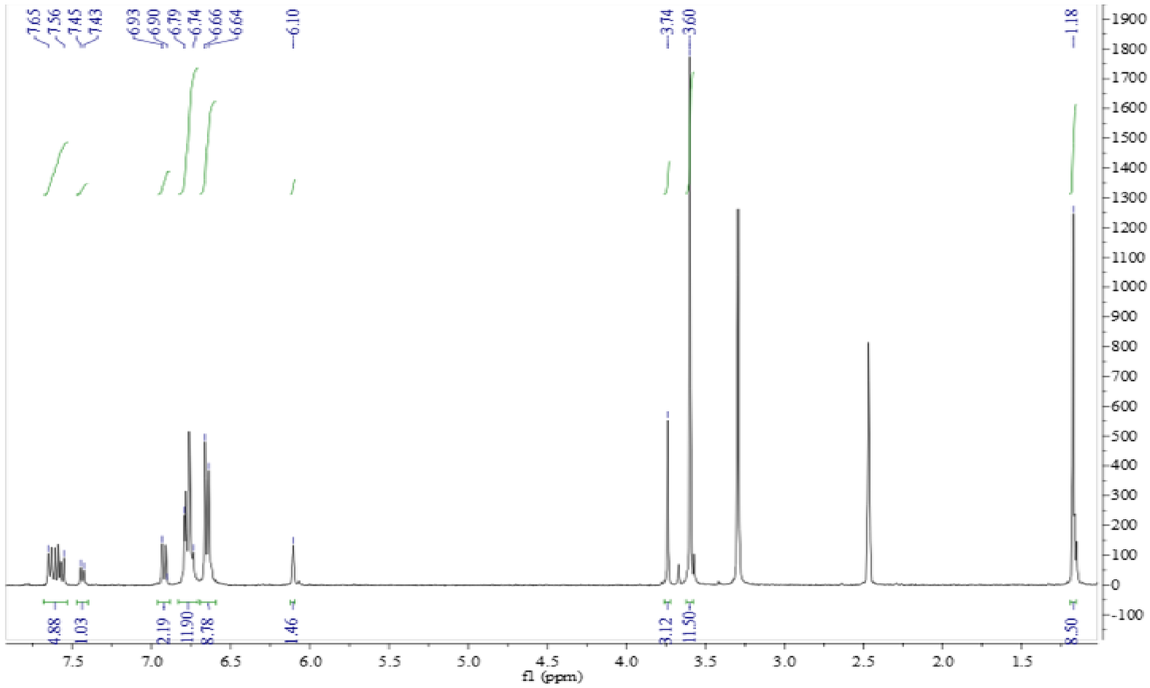

(b)

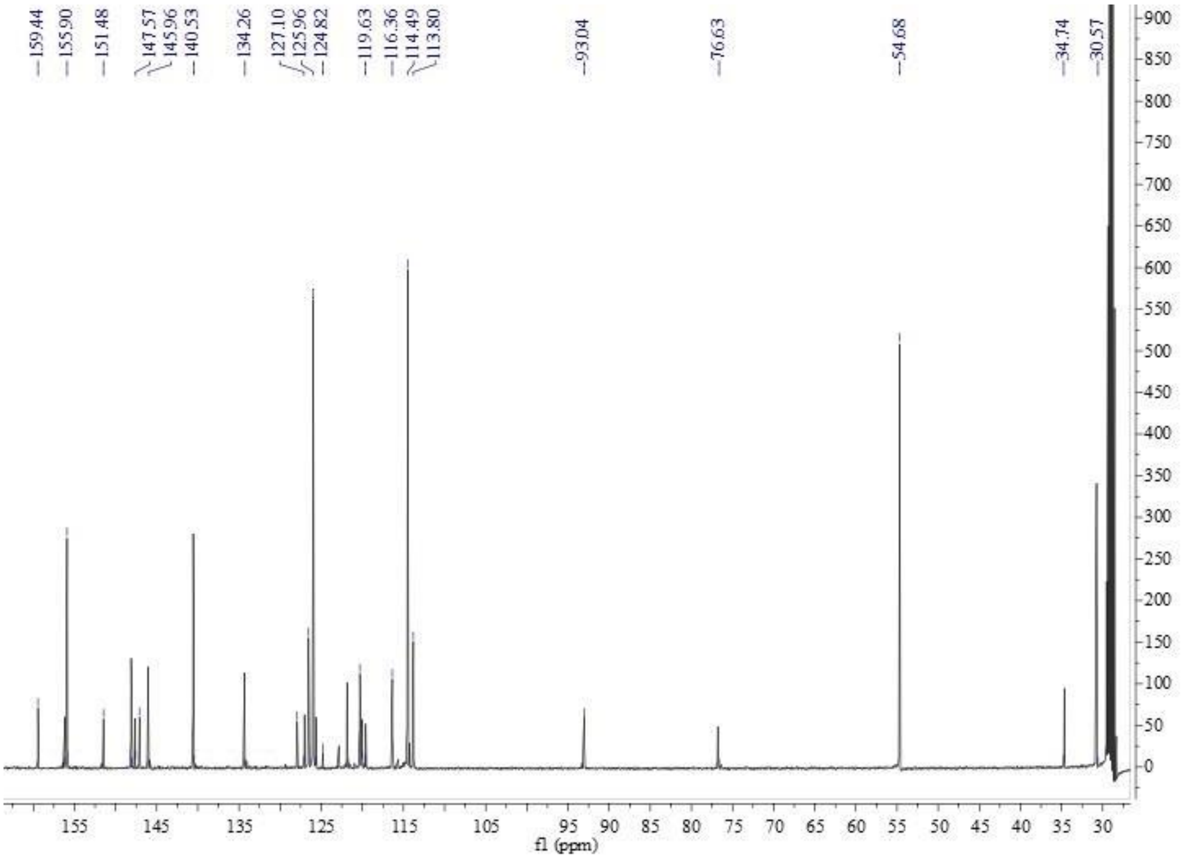

Figure S6. (a)^{1}H-NMR spectrum and (b)^{13}C-NMR spectrum of WY-2 recorded in DMSO-d_6 and acetone-d_6 at RT, respectively.

(a)

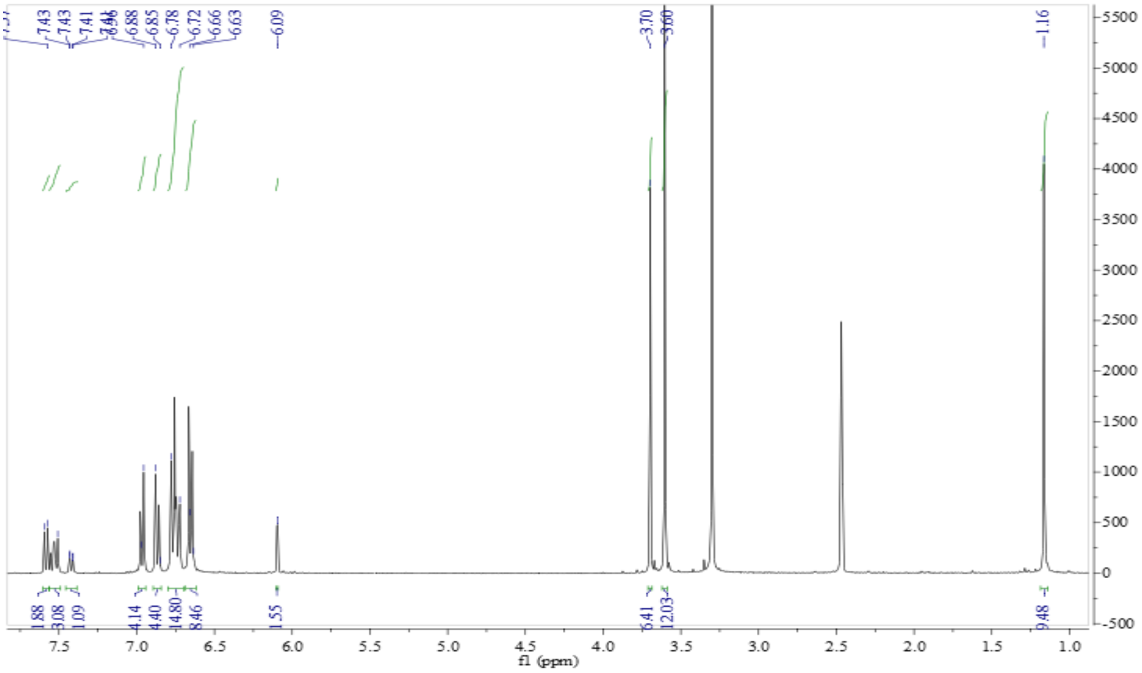

(b)

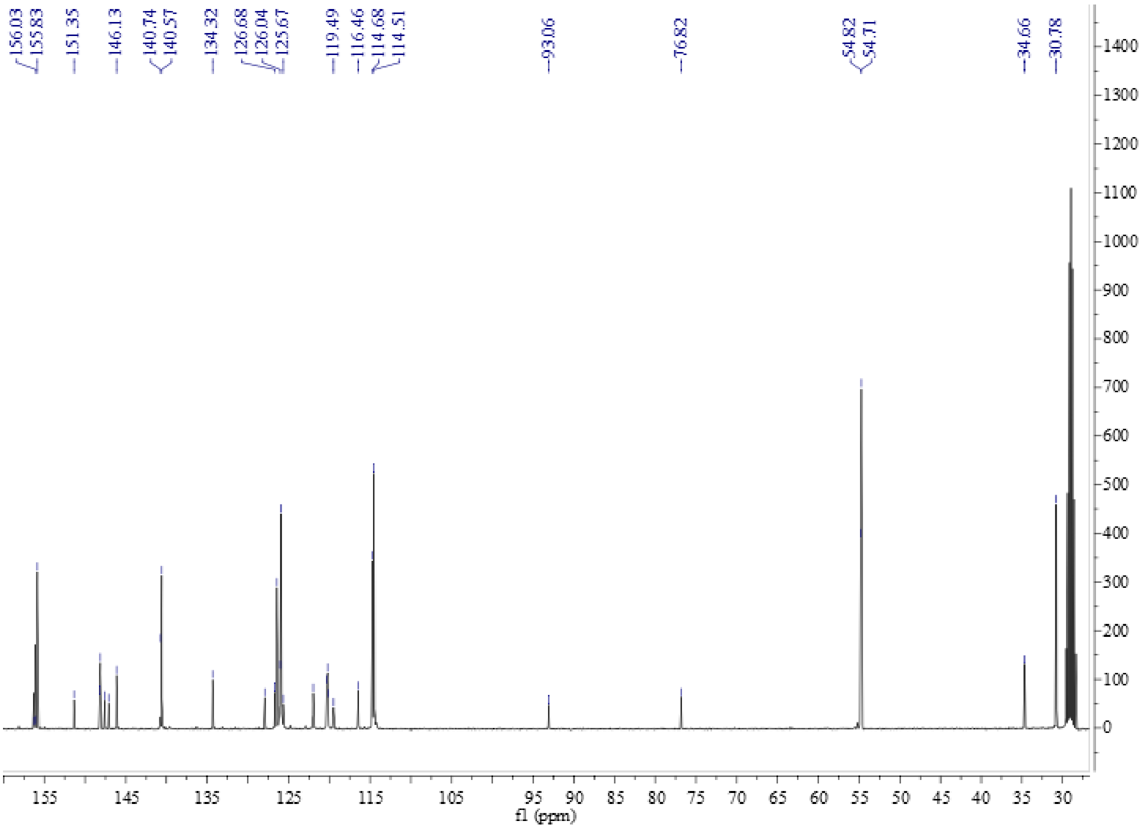

Figure S7. (a)^{1}H-NMR spectrum and (b)^{13}C-NMR spectrum of WY-3 recorded in DMSO-d_6 and acetone-d_6 at RT, respectively.
